# An immune infiltration-based risk scoring system for prognostic stratification in colorectal adenocarcinoma

**DOI:** 10.3332/ecancer.2025.1982

**Published:** 2025-09-03

**Authors:** Oluwafemi Ogundarea

**Affiliations:** Department of Medicine and Surgery, University of Ibadan, Ibadan 200001, Nigeria; a https://orcid.org/0009-0008-2654-7238

**Keywords:** colorectal adenocarcinoma, immune infiltration, prognostic model, CCL8, TYR, risk score, survival analysis

## Abstract

**Background:**

Colorectal adenocarcinoma (CRC) remains a leading cause of cancer-related mortality worldwide, with variable patient outcomes despite treatment advances. Traditional prognostic methods based on clinicopathological variables alone do not fully capture the biological complexity of the disease. This study aims to develop a risk scoring system based on genes associated with tumour-infiltrating immune cells (TIIC-associated genes) to improve prognostic assessment in CRC.

**Methods:**

RNA-seq gene expression and clinicopathological data from The Cancer Genome Atlas Colorectal Adenocarcinoma (TCGA-CRC) database (647 tumour samples, 51 normal tissues) were analysed to identify differentially expressed TIIC-associated genes through comparison with the CIBERSORTx database. Univariate and multivariate Cox analyses were performed to screen for prognostic markers. A Gaussian mixture model was applied to cluster prognostic models and select the model with the most robust gene combination. The resulting risk scoring system was validated in an external cohort (GSE39582) and integrated with clinicopathological variables to develop a prognostic nomogram.

**Results:**

From 128 TIIC-associated genes, an optimal prognostic model comprising CCL8 and Tyrosinase (TYR) was identified. The risk score was calculated as 0.152 × Exp(CCL8)–0.516 × Exp(TYR). Kaplan-Meier analysis confirmed significant survival differences between high-risk and low-risk groups in both TCGA-CRC and GSE39582 (*p* < 0.05). Time-dependent receiver operating characteristic analysis showed area under the curve (AUC) values ranging from 0.605 to 0.696 for 1-, 3- and 5-year survival in TCGA-CRC and GSE39582. Multivariate Cox analysis identified tumour (T stage), node (N stage) and risk score as independent prognostic factors.

**Conclusion:**

Our risk scoring system based on CCL8 and TYR effectively stratifies CRC patients into distinct prognostic groups and could guide treatment decisions, particularly when integrated with TNM staging in a nomogram.

## Background

Colorectal adenocarcinoma (CRC) is a malignant tumour arising from the epithelial lining of the colon and rectum and represents the most common histological subtype of colorectal cancer [1]. It remains one of the leading causes of cancer-related mortality worldwide, with a 5-year survival rate ranging from 91% for early-stage disease to 14% for metastatic disease [2]. Although advancements in surgery, chemotherapy, immunotherapy and targeted therapies have expanded and improved treatment options, patient outcomes remain highly variable [3].

Immune infiltration is central to the tumour microenvironment, influencing tumour progression, metastasis and treatment response [4]. Studies have shown that genes associated with tumour-infiltrating immune cells (TIIC-associated genes) drive tumourigenesis and determine clinical outcomes in CRC [5, 6]. Thus, there is a need for prognostic models that incorporate immune-related molecular features to stratify patients and guide treatment decisions.

Traditional prognostic assessments rely on clinicopathological variables such as tumour size, lymph node involvement and histological grade, which, while informative, do not fully capture the biological complexity of the disease [7]. Gene expression-based prognostic models offer a more targeted approach by leveraging transcriptomic data to predict patient outcomes [8]. Using mathematical modeling techniques such as Cox regression and Gaussian mixture modeling, these models can identify key prognostic genes and stratify patients into distinct risk groups [9].

In this study, we developed a risk scoring system based on TIIC-associated genes to improve prognostic assessment in CRC. Using transcriptomic data from the TCGA-CRC cohort, we identified differentially expressed TIIC-associated genes and screened for prognostic markers using univariate and multivariate Cox regression analyses. A Gaussian mixture model (GMM) was applied to cluster prognostic models and select the most robust gene combination. This approach identified an optimal prognostic model comprising CCL8 and Tyrosinase (TYR), which was used to construct a risk scoring system. Kaplan-Meier survival analysis confirmed that the risk scoring system effectively stratifies patients into high- and low-risk groups, with significant survival differences.

## Methods

### Acquisition of TIIC-associated genes

A dataset of TIIC-associated genes was obtained from the CIBERSORTx database (https://cibersortx.stanford.edu/), which provides a leukocyte gene signature matrix (LM22) consisting of 547 genes across 22 immune cell types. These include natural killer cells, T cells, naïve B cells, memory B cells, plasma cells, monocytes, macrophages, dendritic cells, mast cells, eosinophils and neutrophils.

## Data collection and processing

RNA-seq gene expression and clinicopathological data for 647 CRC solid tumour samples and 51 normal tissue samples (TCGA-CRC) were obtained from the Pan-Cancer Atlas (https://portal.gdc.cancer.gov/). The sequencing reads were mapped to the GRCh38 human genome assembly, with each sample containing expression profiles for 60,660 genes. TCGA-CRC count data was normalised using variance stabilising transformation to ensure uniform variance across genes with different expression levels. For external validation, the GSE39582 dataset was retrieved from the GEO database (https://www.ncbi.nlm.nih.gov/geo/). Normalisation of GSE39582 was performed using the robust multi-array average algorithm, followed by batch effect correction using the ComBat algorithm.

### Identification of differentially expressed genes (DEGs) and TIIC-associated genes in TCGA-CRC cohort

DEGs between tumour and normal tissue samples in TCGA-CRC were identified using DESeq2, with an adjusted* p*-value threshold of <0.05 and a log fold change >1.5. A Venn diagram was used to determine the overlap between DEGs in TCGA-CRC and the 547 TIIC-associated genes. A total of 128 TIIC-associated genes were identified for downstream analysis. Gene expression patterns of the 128 genes were visualised using a heatmap generated with the pheatmap package (version 1.0.12) in R. Functional enrichment analysis was performed using the clusterProfiler (version 4.0), org.Hs.eg.db (version 3.5.0) and GOplot (version 1.0.2) packages in R.

### Screening for prognosis-related TIIC-associated genes

Univariate Cox analysis was performed on the 128 TIIC-associated genes to screen for the most significant prognostic genes, using a *p*-value threshold of <0.05.

### Multivariate Cox analysis and GMM

The selected prognostic genes were then used to construct multiple prognostic models using multivariate Cox analysis. Various combinations of these genes were tested, and the resulting models were clustered using GMM, a probabilistic approach that represents data as a combination of multiple Gaussian distributions. The model with the highest AUC score within any of the clusters was selected as the optimal prognostic model.

### Construction of risk scoring system

A risk scoring system was constructed using the genes from the optimal prognostic model. The risk score was calculated as a weighted sum of the expression levels of these genes, with their respective coefficients from the multivariate Cox analysis serving as weights.

### Evaluation of risk scoring system

Patients in the TCGA-CRC cohort were stratified into high-risk and low-risk groups based on the optimal Kaplan-Meier cutoff of the risk score. The predictive performance of the risk scoring system was assessed using time-dependent receiver operating characteristic (ROC) curves at 1-, 3- and 5-year intervals. This analysis was conducted using the tROC package (version 0.4) in R.

### External validation

The risk scoring system was also externally validated on GSE39582 to further evaluate its performance.

### Identification of independent prognostic factors

Univariate and multivariate Cox analyses were performed on clinicopathological variables, including gender, TNM staging (TNM_T, TNM_N, TNM_M) and the risk score to evaluate their association with survival outcomes and determine whether they can serve as independent prognostic factors.

## Construction and verification of a nomogram

All independent prognostic factors identified through multivariate Cox analysis were used to construct a nomogram for predicting survival, which was evaluated using calibration analysis and decision curve analysis.

## Results

### Identification of TIIC-associated genes in TCGA-CRC cohort

**Figure 1.**
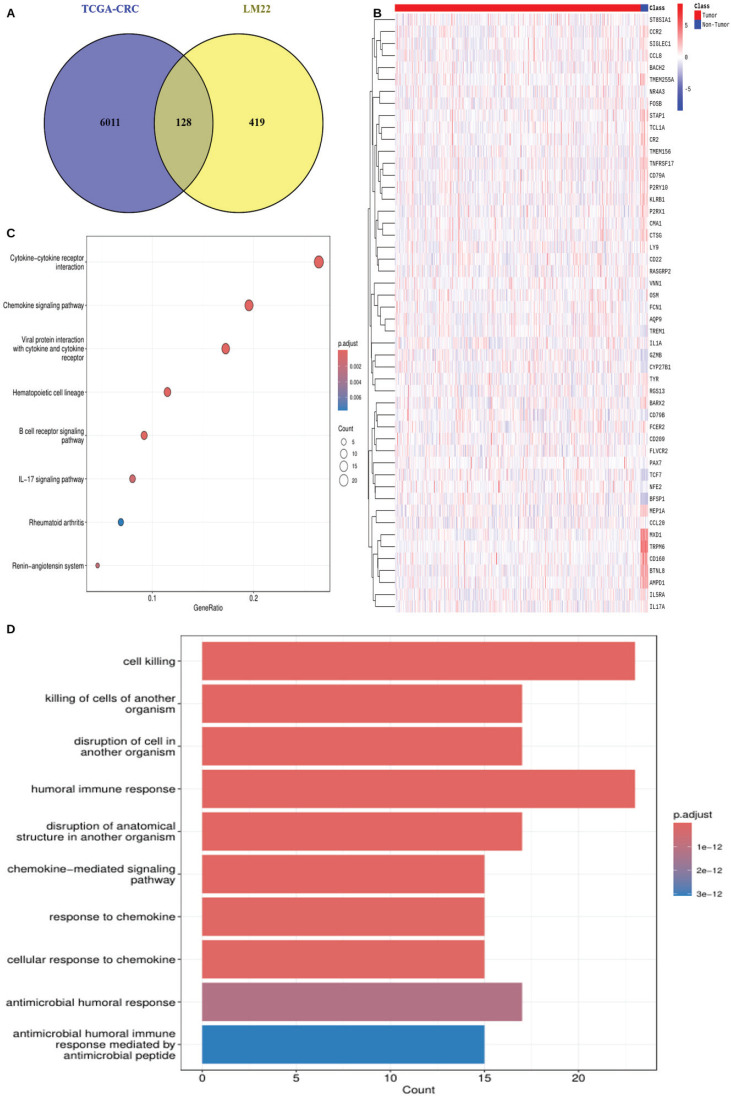
Identification and functional enrichment analysis of TIIC-associated genes in TCGA-CRC cohort. (a): Venn diagram showing the intersection between DEGs and TIIC-associated genes. (b): Heatmap of first 50 of 128 TIIC-associated genes in GSE39582. (c): GO analysis. (d): KEGG enrichment analysis.

Using DESeq2, a total of 6,011 DEGs were identified between 647 CRC tumour samples and 51 normal tissue samples in TCGA-CRC, based on an adjusted *p*-value <0.05 and log fold change >1.5. A Venn diagram analysis showed 128 overlapping TIIC-associated genes between the 6,011 DEGs and the 547 TIIC-associated genes obtained from the CIBERSORTx database, which were subsequently used for downstream analysis (Figure 1a). To validate these findings, the expression patterns of the 128 TIIC-associated genes were examined in the external dataset GSE39582 (Figure 1b). Functional enrichment analysis was then performed to explore the biological significance of these genes. KEGG pathway enrichment analysis showed significant enrichment in pathways such as cytokine-cytokine receptor interaction, chemokine signaling, viral protein interaction with cytokines and receptors, hematopoietic cell lineage and B cell receptor signaling (Figure 1c). Gene ontology (GO) analysis showed that processes related to cell killing, humoral immune response and chemokine-mediated signaling were significantly enriched (Figure 1d).

### Identification of prognosis related TIIC-associated genes

Univariate Cox analysis identified seven candidate prognostic genes (Table 1), which were selected for risk score modeling.

### Multivariate Cox analysis and GMM

A multivariate Cox analysis was performed on various combinations of the 7 prognostic TIIC-associated genes, generating a total of 127 prognostic models. These models were then clustered using GMM, which grouped them into five distinct clusters based on their characteristics. The optimal prognostic model was identified in cluster three and achieved the highest AUC score. This model included two genes, CCL8 and TYR. The coefficients and hazard ratios of this model are presented in Table 2.

### Construction of the risk scoring system

A risk scoring system for predicting the prognosis of CRC patients was constructed based on the expressions of CCL8 and TYR. Using the coefficients from the multivariate Cox analysis, the risk score was calculated as follows:

Risk score = 0.152 × Exp(CCL8) – 0.516 × Exp(TYR)

Annotation: Exp Expression value

### Evaluation of risk scoring system

**Figure 2.**
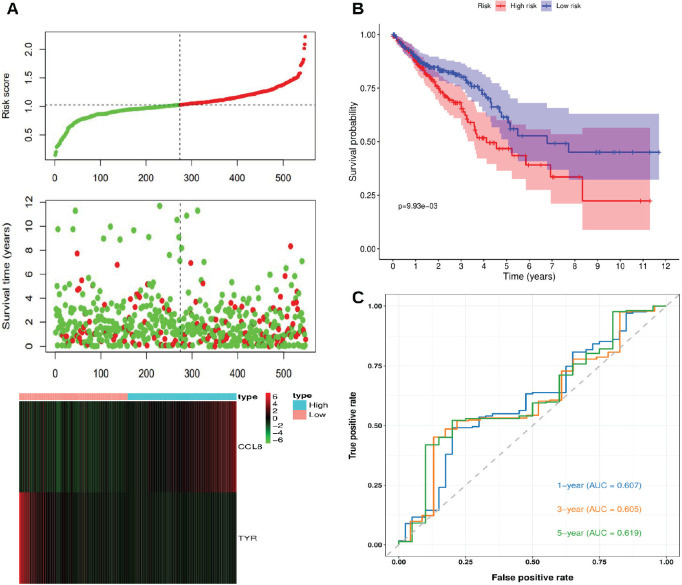
Risk score analysis, survival analysis and prognostic performance of the risk scoring system in TCGA-CRC. (a): Risk scores, survival times and expression levels of CCL8 and TYR. (b): Kaplan-Meier analysis of overall survival between high-risk and low-risk groups. (c): Time-dependent ROC curves for 1-, 3- and 5-year survival.

Patients in the TCGA-CRC cohort were stratified into high-risk and low-risk groups based on the optimal Kaplan-Meier cutoff of the risk score. Figure 2a illustrates the relationships between risk scores, patient survival times and the expression levels of CCL8 and TYR. The risk plot suggests that high-risk patients generally exhibit shorter survival times and poorer prognoses compared to low-risk patients. Also, higher CCL8 expression is associated with increased risk scores, while higher TYR expression is associated with lower risk scores. Figure 2b shows a statistically significant difference in survival between high-risk and low-risk patients (*p* < 0.05), with low-risk patients demonstrating higher survival probabilities and longer overall survival times. Figure 2c presents the time-dependent ROC curves, with AUC values of 0.607, 0.605 and 0.619 for 1-, 3- and 5-year survival, respectively.

### External validation

**Figure 3.**
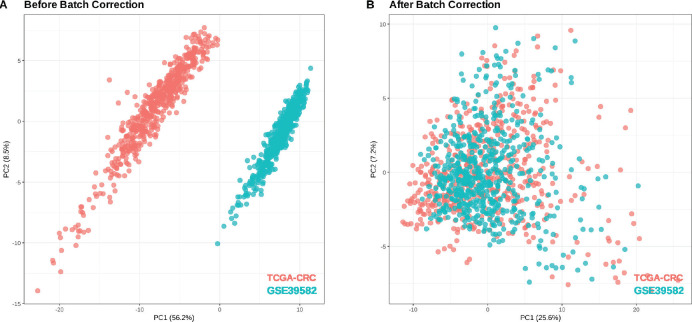
PCA plots for samples in TCGA-CRC and GSE39582. (a): Before batch correction. (b): After batch correction.

GSE39582 (566 tumour samples, 19 normal tissues) was used for external validation of the risk scoring system. Batch effects from technical variations (e.g., sequencing platforms, sample processing, RNA extraction methods, reagent lots, storage conditions and operator handling) were corrected using the Combat algorithm, with TCGA-CRC as the reference. Before correction (Figure 3a), Principal component analysis (PCA) plots showed distinct separation due to batch effects. After correction (Figure 3b), variance was reduced and the datasets followed a more similar distribution.

**Figure 4.**
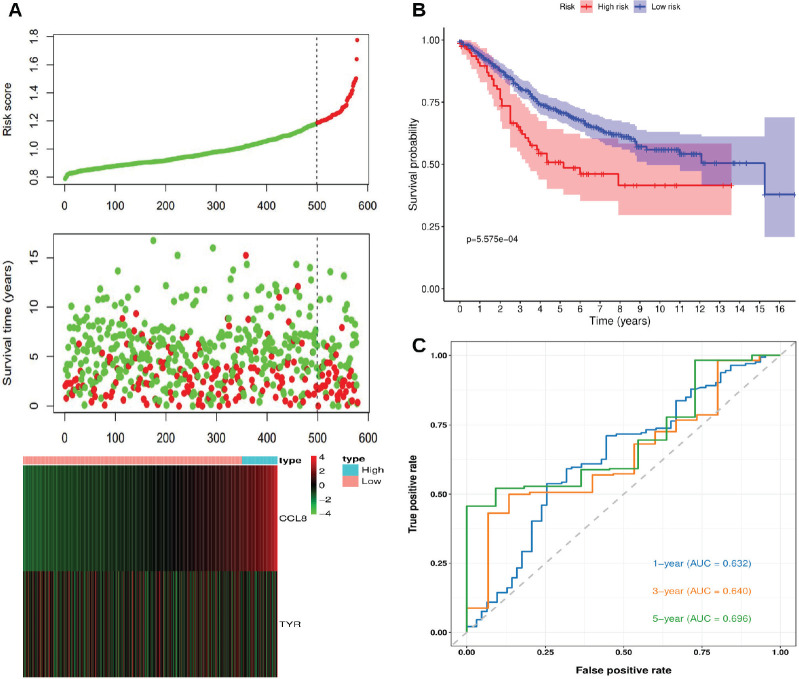
Risk score analysis, survival analysis and prognostic performance of the risk scoring system in GSE39582. (a): Risk scores, survival times and expression levels of CCL8 and TYR. (b): Kaplan-Meier analysis of overall survival between high-risk and low-risk groups. (c): Time-dependent ROC curves for 1-, 3- and 5-year survival.

The risk score was calculated for each patient using the same risk scoring formula. Patients were stratified into high-risk and low-risk groups based on the optimal Kaplan-Meier cutoff of the risk score. Figure 4a shows the relationships between risk scores, patient survival times and the expression levels of CCL8 and TYR. The risk plot indicates that high-risk patients have shorter survival times and poorer prognoses compared to low-risk patients. Also, CCL8 expression is positively correlated with the risk score, whereas TYR expression is negatively correlated. Figure 4b confirmed a statistically significant survival difference between high-risk and low-risk patients (*p* < 0.05), with low-risk patients exhibiting higher survival probabilities and longer overall survival times. Figure 4c displays the time-dependent ROC curves, with AUC values of 0.632, 0.640 and 0.696 for 1-, 3- and 5-year survival, respectively.

### Identification of independent prognostic factors

**Figure 5.**
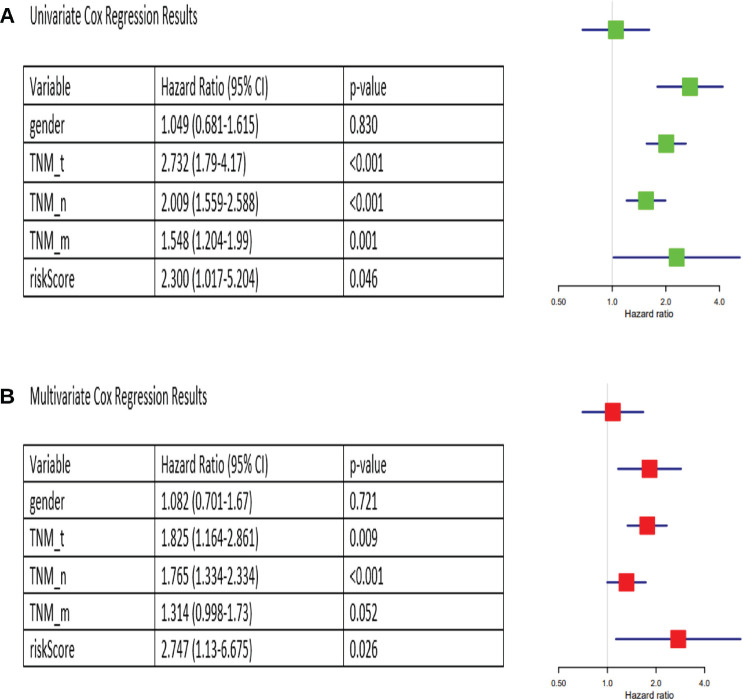
Univariate and multivariate Cox analyses for identification of independent prognostic factors. (a): Univariate Cox results. (b): Multivariate Cox results.

Univariate Cox analysis found TNM_T, TNM_N, TNM_M and risk score to be significantly associated with survival (*p* < 0.05), whereas gender was not (Figure 5a). Multivariate Cox analysis identified TNM_T, TNM_N and risk score as independent prognostic factors for CRC (*p* < 0.05), while TNM_M was not independent and could be represented by other clinicopathological variables (Figure 5b).

### Construction and verification of a nomogram

**Figure 6.**
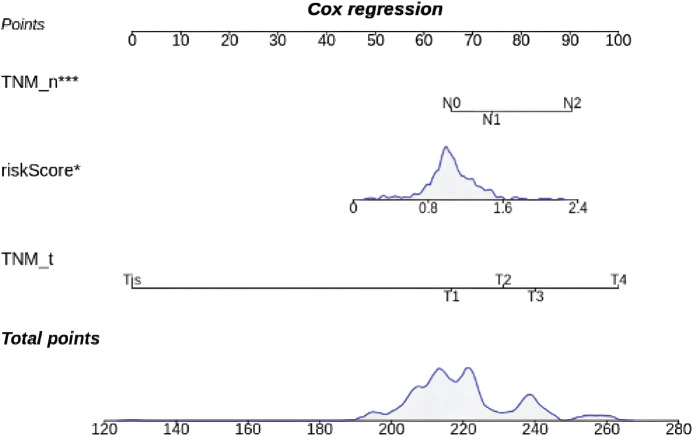
Prognostic nomogram.

TNM_T, TNM_N and risk score were incorporated into a nomogram (Figure 6).

A nomogram integrating TNM_T, TNM_N and risk score to estimate prognosis. Higher total points indicate a greater risk of poor survival outcomes.

**Figure 7.**
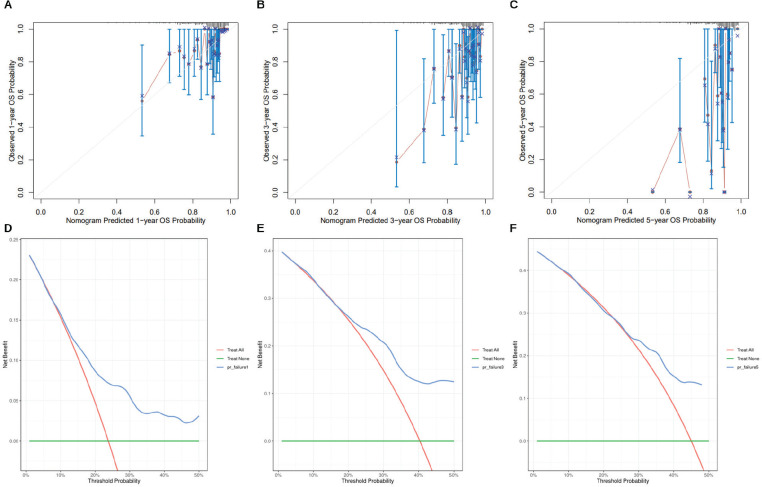
Calibration analysis and decision curve analysis of the nomogram. (a–c): Calibration curves for 1-, 3- and 5-year survival. The x-axis represents the predicted overall survival probability, while the y-axis shows the actual observed survival. The 45° gray line represents the ideal prediction. (d–f): Decision curve analysis for 1-, 3- and 5-year survival, showing the clinical net benefit at different threshold probabilities.

The 1-, 3- and 5-year overall survival predictions of the nomogram were evaluated using calibration analysis (Figure 7a–c) and decision curve analysis (Figure 7d–f). The results showed that the nomogram demonstrated favourable prognostic performance.

## Discussion

In this study, we established a novel prognostic risk scoring system for CRC based on two TIIC-associated genes, CCL8 and TYR. Our findings demonstrate that this risk scoring system effectively stratifies patients into high-risk and low-risk groups with significantly different survival outcomes, both in the TCGA-CRC cohort and in GSE39582, the external validation dataset. We also showed that the risk score, together with TNM_T and TNM_N, serves as an independent prognostic factor, allowing for the construction of a nomogram with favourable prognostic performance.

The optimal prognostic model identified through our multivariate Cox analysis and GMM comprises two genes with opposing effects on patient prognosis, namely CCL8 and TYR. CCL8 (C-C Motif Chemokine Ligand 8) showed a positive coefficient of 0.152 in our model, indicating that higher expression is associated with increased risk and poorer outcomes. In contrast, TYR exhibited a negative coefficient of −0.516, suggesting that elevated expression correlates with reduced risk and improved survival.

CCL8, also known as monocyte chemoattractant protein-2, is a chemokine that attracts monocytes, lymphocytes, basophils and eosinophils by interacting with several chemokine receptors [10]. Its role in cancer progression has been well documented. Studies have shown that CCL8 promotes breast cancer dissemination, enhances the migration and invasion of esophageal cancer cells and drives lung cancer progression [11–14]. Interestingly, CCL8 has also been reported to inhibit melanoma metastasis, suggesting that its function may be highly variable across cancer types [15]. In colorectal cancer specifically, elevated CCL8 expression has been linked to increased tumour cell invasion and migration [16, 17]. Our findings align with these observations in epithelial cancers, as we found that higher CCL8 expression correlates with increased risk scores and poorer survival outcomes.

TYR encodes an enzyme that targets melanosomes in melanocytes and serves as a rate-limiting enzyme in melanin synthesis [18, 19]. While primarily known for its role in pigmentation, recent evidence suggests that TYR may also contribute to immune regulation and oxidative stress response [20, 21]. In our study, TYR expression was negatively correlated with risk scores, indicating a potential protective role in CRC. Interestingly, although TYR itself appears to have a tumour-suppressive effect, TYR kinase, which phosphorylates it, has been previously implicated in the development of adenomatous polyps, ulcerative colitis and CRC [22]. This finding warrants further investigation into the mechanisms by which TYR might influence tumour suppression or immune surveillance in colorectal cancer.

Traditional prognostic assessment for CRC relies heavily on the TNM staging system, which, while informative, does not fully capture the biological heterogeneity of the disease [7]. Several gene expression-based prognostic signatures have been proposed for colorectal cancer, including the Oncotype DX Colon Cancer Assay (12-gene panel) and ColoPrint (18-gene panel) [23, 24]. However, these signatures often require larger gene sets and may not specifically incorporate immune-related genes.

Our risk scoring system uses only two genes, making it more cost-effective and clinically applicable. Clinicians can assess patient prognosis and make informed decisions by simply calculating the risk score. It is also based on TIIC-associated genes, which are involved in the tumour microenvironment and recognised as drivers of cancer progression [5, 6].

The risk scoring system may have meaningful clinical applications. By stratifying patients into high-risk and low-risk groups, it could guide treatment decisions. High-risk patients may benefit from more aggressive treatment and closer monitoring, while low-risk patients may be spared unnecessary interventions and their associated toxicities. Also, integrating our risk score with established clinicopathological variables (TNM_T and TNM_N) in a nomogram provides a valuable tool for personalised risk assessment. Beyond prognosis, CCL8 and TYR may also represent novel therapeutic targets in CRC.

Our study has several limitations. First, while the risk scoring system demonstrated significant prognostic value, the AUC values (ranging from 0.605 to 0.696) suggest that predictive accuracy could be improved. Future studies should explore integrating additional molecular features, such as DNA methylation or lncRNA expression, to improve the model performance. Second, our study was based on retrospective cohorts, which may have introduced selection bias. Prospective validation in clinical trials would provide stronger evidence for the clinical utility of our risk scoring system. *In vivo* and *in vitro* functional studies are also needed to better understand the precise mechanisms by which CCL8 and TYR influence CRC progression.

Moreover, while our study focused on overall survival, future research could assess the prognostic value of our model for other important clinical endpoints, such as disease-free survival, progression-free survival or response to specific therapeutic regimens. Finally, the heterogeneity of CRC, including differences between right-sided and left-sided tumours and various molecular subtypes (e.g., microsatellite instability status, CpG island methylator phenotype), was not fully addressed in our analysis. Investigating how our risk scoring system performs across these different subgroups could identify subtype-specific prognostic patterns.

## Conclusion

In summary, we have developed and validated a novel risk scoring system based on two TIIC-associated genes, CCL8 and TYR, that effectively stratifies CRC patients into distinct prognostic groups. The risk score, together with clinicopathological variables, serves as an independent prognostic factor and can be integrated into a nomogram for comprehensive risk assessment. Further validation is needed to evaluate the robustness of this system, while functional studies could clarify the role of CCL8 and TYR in CRC.

## Conflicts of interest

OO holds a patent unrelated to the subject matter of this manuscript. This does not influence the content or conclusions of the paper.

## Funding

The author declare that they have not received any funding for this research.

## Informed consent

Not applicable.

## Availability of data and materials

The datasets generated and/or analysed during the current study are available in the TCGA (https://portal.gdc.cancer.gov/), GEO (https://www.ncbi.nlm.nih.gov/geo/) and CIBERSORTx (https://cibersortx.stanford.edu/) databases.

## Author contributions

OO confirms being the sole contributor to this work and is responsible for all aspects of the study, including conception, design, analysis, interpretation and manuscript preparation.

## Figures and Tables

**Table 1.** Seven prognostic TIIC-associated genes identified by univariate Cox analysis.

Gene	Full name	HR (95% CI)
GNG7	G protein subunit gamma 7	1.266 (1.059–1.515)
SIGLEC1	Sialic acid binding Ig like lectin 1	1.170 (1.026–1.334)
RASGRP2	RAS guanyl releasing protein 2	1.245 (1.020–1.520)
CXCL3	C-X-C motif chemokine ligand 3	0.866 (0.756–0.992)
BACH2	BTB and CNC homology 2	1.247 (1.011–1.537)
CCL8	C-C motif chemokine ligand 8	1.175 (1.006–1.371)
TYR	Tyrosinase	0.587 (0.349–0.987)

Annotation: HR, Hazard Ratio; 95% CI 95%, Confidence Interval

**Table 2.** The coefficient and hazard ratios of CCL8 and TYR in the optimal multivariate Cox model.

Gene	Coefficient	Hazard ratio	Lower 95% CI	Upper 95% CI
CCL8	0.152	1.164	0.998	1.359
TYR	−0.516	0.597	0.355	1.003

Annotation: 95% CI 95%, Confidence Interval
